# Influence of prenatal exercise on the relationship between maternal overweight and obesity and select delivery outcomes

**DOI:** 10.1038/s41598-022-22283-0

**Published:** 2022-10-15

**Authors:** Samantha M. McDonald, Steven Mouro, Breanna Wisseman, Christy Isler, James DeVente, Edward Newton, Jason Hildebrand, Devon Kuehn, George Kelley, Lisa Chasan-Taber, Nicholas T. Broskey, Linda E. May

**Affiliations:** 1 School of Kinesiology and Recreation, Illinois State University, Normal, IL USA; 2 Campbell University School of Osteopathic Medicine, Lillington, NC USA; 3 Department of Obstetrics and Gynecology, ECU, 1851 MacGregor Downs Rd, MS#701, Greenville, NC 27834 USA; 4 Human Performance Laboratory, East Carolina University (ECU), Greenville, NC USA; 5 Department of Pediatrics, ECU, Greenville, NC USA; 6 Department of Epidemiology and Biostatistics, West Virginia University, Morgantown, WV USA; 7 Department of Biostatistics and Epidemiology, University of Massachusetts, Amherst, MA USA; 8 East Carolina Diabetes and Obesity Institute, ECU, Greenville, NC USA; 9 Department of Foundational Sciences and Research, ECU, Greenville, NC USA

**Keywords:** Physiology, Health care, Medical research, Risk factors

## Abstract

Women with overweight or obesity (OWOB) have an increased risk of cesarean birth, preterm birth (PTB), and high birth weight infants. Although regular exercise decreases this risk in healthy weight women, these associations have not been explored in OWOB. Women were randomized at 13–16 weeks’ gestation to 150-min of moderate-intensity exercise (n = 131) or non-exercising control (n = 61). Delivery mode, gestational age (GA), and birth weight (BW) were obtained via electronic health records. Pregnant exercisers had no differences in risk of cesarean birth, PTB, or BW compared to control participants. OWOB exercisers had higher rates of cesarean birth (27.1% vs. 11.1%), trends of higher PTB (15.3% vs. 5.6%), but normal weight babies relative to normal weight exercisers. Controlling for race and body mass index (BMI), maternal exercise reduced the relative risk (RR) for cesarean birth from 1.63 to 1.43. Cesarean births predicted by pre-pregnancy BMI and fitness level, whereas BW was predicted by race, gestational weight gain (GWG), pre-pregnancy fitness level, and exercise level. Cesarean birth was predicted by pre-pregnancy BMI and fitness level, while maternal exercise reduced the magnitudes of the relative risks of cesarean birth. Maternal exercise, pre-pregnancy fitness level, and GWG predict neonatal BW.

**Trial Registration:** Influence of Maternal Exercise on Infant Skeletal Muscle and Metabolomics-#NCT03838146, 12/02/2019, https://register.clinicaltrials.gov/prs/app/template/EditRecord.vm?epmode=Edit&listmode=Edit&uid=U0003Z0X&ts=8&sid=S0008FWJ&cx=77ud1i.

## Introduction

Every year in the United States, nearly one-third of pregnant women deliver their infants via cesarean birth, accounting for more than 1.3 million deliveries^1^. Complications during labor, such as arrest, maternal or fetal distress, and abnormalities of the placenta or umbilical cord, often result in cesarean birth. Consequently, both mother and neonate face increased risk of various adverse health outcomes including postpartum hemorrhage, shoulder dystocia, fetal injury, and respiratory distress associated with cesarean birth. Women with obesity are more likely to have increased risk of poor pregnancy outcomes such as intrapartum complications, macrosomia, small or large for gestational age birth weight, placing them at a higher risk for cesarean birth^1,2^.

The American College of Obstetrics and Gynecology (ACOG) recommends 150-min of moderate physical activity per week during pregnancy and postpartum period due to growing evidence demonstrating improved pregnancy outcomes due to exercise during pregnancy^3^. Research has shown that physical activity during pregnancy is associated with decreased risk of gestational diabetes, postpartum depression symptoms, preeclampsia, and with more women achieving adequate weight gain^4,5^. It has also been shown that neonates exposed to exercise in utero exhibit lower percent body fat^6^, and normalized birth weight^7^. Similarly, antenatal exercise has resulted in fewer complications during labor and delivery, with a quicker recovery time compared to inactive counterparts^5^. A meta-analysis of randomized control trials reported that exercise during pregnancy increased the likelihood of a normal delivery for normal weight women^8^. In addition, a randomized controlled trial demonstrated reduced rates of cesarean birth in overweight women that exercised during pregnancy^9^. However, the intervention was only 12–14 weeks of gestation. In addition, the effect of supervised prenatal exercise at ACOG recommended levels throughout multiple trimesters on delivery mode and pregnancy outcomes for overweight and obese women is unknown.

While data suggests that physical activity during pregnancy improves maternal and neonatal outcomes, the underlying physiological mechanisms are not well understood. Prenatal exercise is postulated to reduce inflammation^10,11^, which can result from obesity, thus mediating the positive impact on the intrauterine environment, placental growth and function, and maternal–fetal health. Therefore, if prenatal exercise reduces this inflammatory state, this will result in lower risk of adverse delivery outcomes and rates of cesarean birth. Unfortunately, current data on exercise and mode of delivery in overweight or obese (OWOB) pregnant women is limited. Thus, the purpose of this study was to assess the influence of antenatal exercise on the association between maternal BMI, cesarean birth, preterm birth, and neonatal birth weight. We hypothesized that antenatal exercise would decrease primary cesarean occurrence and preterm births; further, we hypothesized maternal exercise would be associated with normal range birth weights in overweight and obese women, similar to pregnancy outcomes in normal weight women.

## Methods

### Study design

This study is a secondary analysis of a single-blinded, four-arm randomized controlled exercise intervention trial that focused on determining the influence of different modes of maternal exercise during pregnancy on neonatal health outcomes^12^. Pregnant women were randomized to one of four intervention groups: aerobic training, resistance training, combination (resistance and aerobic) training and non-exercising control group. The study was 24+ weeks in duration, following women from the early 2nd trimester (13–16 weeks of gestation) until delivery. All protocols were approved by the East Carolina University Institutional Review Board. The study was conducted in accordance with the Declaration of Helsinki, and approved by the Institutional Review Board of East Carolina University (#12-002524); the study was registered on clinicaltrial.gov #NCT03838146, on 12/02/2019. Written informed consent was obtained from all participants as well as a clearance letter from their obstetric provider confirming their pregnancy and ability to engage in moderate-intensity exercise.

### Study population

Healthy women with a low-risk, singleton pregnancy was recruited from local obstetric clinics via fliers and email announcements. Women were eligible for the study if they met the following criteria: gestational age ≤ 16 weeks, ages 18–40 years, pre-pregnancy body mass index (BMI) of 18.50–34.99 kg m^−2^, not currently using alcohol, tobacco, recreational drugs, or medications for chronic disorders, no contraindications to exercise in pregnancy as outlined by the American College of Obstetricians and Gynecologists, with no pre-existing diabetes, hypertension, or other cardiovascular disease. Women diagnosed with gestational diabetes during the study were still included in the intervention.

### Pre-intervention exercise testing

Prior to randomization, all participants completed a validated submaximal modified Balke treadmill exercise test^13^ to assess aerobic exercise capacity as well as the one-repetition maximum (1RM) test to assess strength. During the treadmill test, oxygen and carbon dioxide levels were assessed via indirect calorimetry (Parvo Medics, TrueOne 2400, Sandy, UT) to determine aerobic capacity, i.e., fitness level, VO_2peak_ (ml O_2_ kg^−1^ min^−1^) and maternal heart rate (HR), the latter of which was continuously measured (Polar FS2C heart rate monitor). Following the test, target heart rate zones (THR) were determined in order to establish target heart rates (THRs) corresponding to 40–59% VO_2peak_ for moderate-intensity exercise^14^. During the 1RM tests, participants performed resistance exercises based on the American College of Sports Medicine (ACSM) protocol^15^ to determine maximum strength encompassing a full array of upper and lower body exercises on machines, free weights, and body weight. Participants randomized to a group with resistance exercise started at 60% of their 1RM test weight.

### Exercise intervention

Participants engaged in 50 min of moderate-intensity exercise sessions three times per week for 24+ weeks. All exercise sessions were supervised by certified trainers at one of two university-affiliated facilities and followed a standard protocol as described previously^12^. Exercise sessions consisted of a 5-min warmup, 50 min of moderate-intensity (40–59% VO_2_peak) exercise, and a 3–5-min cooldown. Exercise intensity was monitored via maternal HR, using a Polar FS2C heart rate monitor, as well as the 15-point Borg scale of perceived exertion to ensure compliance with the protocol as previously published^16^. The aerobic training group completed moderate intensity training on the treadmill, elliptical, recumbent bicycle, rower and/or stair-stepping equipment. Speed and grade were adjusted on the treadmill, and resistance and speed levels were adjusted on the elliptical and recumbent bike in order to maintain the appropriate HR zone. The resistance training group completed sessions of 2–3 sets of 15 repetitions of each exercise at a moderate intensity^28^. For example, Cybex machines (i.e., leg extension, leg curl, shoulder press, chest press, triceps extension, latissimus dorsi pull down), dumbbells (biceps curls, lateral shoulder raises, front shoulder raises), resistance bands/dumbbells, exercise balls, benches, and/or mats were used. The combination training group performed half of the aerobic protocol and half of the resistance protocol exercises in five circuits lasting 4.5–5 min, each. For the combination group, resistance exercises were performed at 15 repetitions (same exercises and equipment as the resistance group) and the aerobic exercises were performed on the same equipment as the aerobic group. To verify that the proper intensity was achieved during sessions, the Borg scale rating of perceived exertion (RPE)^26,29^, and the “talk test” were used. HR monitoring (Polar FS2C) ensured appropriate target HR ranges were maintained; target HR zones validated for pregnant women were utilized^27^.

The non-exercising control group performed stretching of all muscle groups and breathing exercises with inhalation and exhalation^17,18^. This approach is aimed at maintaining contact and engagement with control participants throughout the study and is critical to retention^19^. This also ensures equal contact between exercisers and control participants, thus attenuating varying participant engagement as a potential covariate. Exercise adherence was tracked for all participants as the number of sessions attended divided by the total sessions possible. Participants completing at least 80% of sessions were considered exercise adherent and included in the per protocol analyses. Participant activities during pregnancy were also quantified as MET∙min∙week^−1^ of exercise based on the calculation of (frequency × duration of session in minutes) then multiplied by the published MET (metabolic equivalent) level for their specific exercise. The totals for all weeks were summed and averaged for the pre-pregnancy MET∙min∙week^−1^ value.

### Maternal covariates

Maternal demographic and pregnancy-related characteristics including age, race/ethnicity, parity, pre-pregnancy weight and height, gestational diabetes mellitus status (yes or no) were abstracted from various sources including pre-screening eligibility questionnaires and electronic health records. Pre-pregnancy BMI was calculated using self-reported height and weight collected from the pre-screening eligibility questionnaire at enrollment via the following established equation^5^:

$$BMI= ((Wt [kg]/({ht [m}^{2}]))$$

BMI classifications used were previously established^20^:18.5–24.9 kg m^−2^ was designated as normal, 25.0–29.9 kg m^−2^ was overweight, and > 30.0 kg m^−2^ was obese. Additionally, gestational weight gain (GWG) was determined by calculating the differences in maternal weight gain between pre-pregnancy weight and delivery weight acquired from electronic health records.

### Pregnancy and delivery outcomes

Delivery mode (spontaneous vaginal delivery or unplanned/emergency or planned cesarean birth, reason for delivery mode), gestational age in weeks, and birth weight in kilograms and infant sex were acquired from electronic health records. Macrosomia was defined as infants with birth weight 4.0 kg or greater. Since our question focused on the potential physiological influence of exercise on the delivery process, 14 women with planned cesarean deliveries (6 exercise, 8 controls) were excluded from the analysis of risk for cesarean birth. Preterm birth was defined as a delivery < 37 weeks of gestation as previously established by medical professionals as well as research showing an association with poorer health outcomes^21^.

### Statistical analysis

Between-group differences in maternal demographic characteristics, pregnancy and delivery outcomes were determined via Student’s t-tests and Pearson Chi-Square tests, where appropriate. Between-group differences were analyzed using intention-to-treat analysis as well as the per-protocol approach based on those who were exercise adherent. Conditional distributions evaluated for the assumptions of linear and Poisson regression were satisfied. Following intention-to-treat, ANCOVA and Poisson regression models were performed to evaluate the effects of prenatal exercise on the association between maternal BMI and pregnancy as well as delivery outcomes. The primary outcomes for this study were: occurrence of non-elective cesarean births, birth weight (kg; continuous), preterm (< 37 or ≥ 37 weeks). The main effects between maternal BMI and the outcomes of interest were assessed first, followed by the effects of prenatal exercise (MET∙min∙week^−1^) to evaluate the potential attenuation of the relationship between maternal BMI and delivery outcomes consequent to prenatal exercise. Where appropriate, the following covariates were then considered: maternal race, age, gestational age, GWG, aerobic capacity, and parity. Statistical analyses were performed using SAS, version 9.4 (Cary, NC). Statistical significance was determined a priori at p < 0.05 with two-tailed analyses.

### Ethics approval and consent to participate

All protocols were approved by the East Carolina University Institutional Review Board. The study was conducted in accordance with the Declaration of Helsinki, and approved by the Institutional Review Board of East Carolina University (#12-002524). Written informed consent was obtained from all participants as well as a clearance letter from their obstetric provider confirming their pregnancy and ability to engage in moderate-intensity exercise.

**Figure 1:**
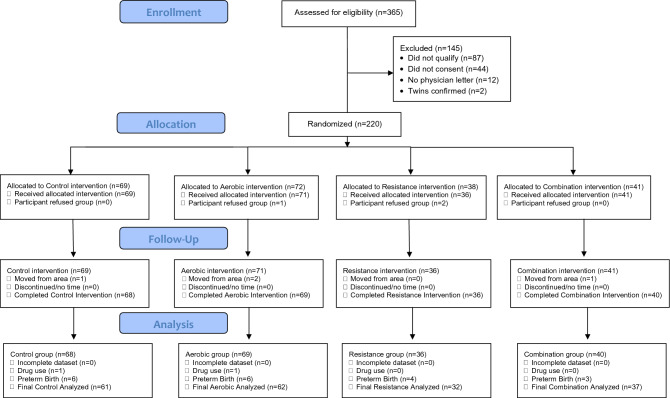
Recruitment and enrollment of pregnant participants.

## Results

We assessed 365 women for eligibility, and were able to recruit and randomize 220 participants (Fig. 1). Of these participants (Fig. 1), we analyzed data from 213 participants (69 aerobic, 36 resistance, 40 combination, 68 controls).

### Descriptive statistics

We found significant between-group differences for maternal race, pre-pregnancy BMI, and aerobic capacity (i.e., fitness level); for example, women in the exercise group were, on average, Caucasian, had a lower pre-pregnancy BMI (25.1 vs 26.8 kg m^−2^) and higher prepregnancy fitness level (23.4 vs 21.6 ml O_2_∙kg^−1^∙min^−1^) relative to all controls (Table 1). Similarly, significant differences were found between BMI groups; for instance, a greater proportion of OWOB pregnant women were non-Hispanic black (23.6 vs 9.9%), and less aerobically fit (20.9 vs 24.8 ml O_2_∙kg^−1^∙min^−1^) at study entry relative to normal weight women (Suppl. Table 1).

**Table 1:** Intention to treat analysis of maternal descriptive, pregnancy, and delivery outcomes by *Intervention Group.*

Characteristics	Exercise (n = 131)	Control (n = 61)	p value
**Demographics**			
Age (year)	30.5 (3.9)	29.4 (4.1)	0.06
Parity	1.0 (1.0, 3.0)	1.0 (1.0, 3.0)	0.65
NH black (%)	12.4	25.4	0.03*
Pre-pregnancy BMI (kg m^−2^)	25.1 (4.3)	26.8 (5.4)	0.03*
Overweight or obese (%)	45.0	57.4	0.12
Aerobic capacity (VO_2peak_)	23.4 (4.7)	21.6 (4.1)	0.01*
**Pregnancy outcomes**			
Gestational weight gain (lbs.)	32.5 (11.8)	30.0 (12.5)	0.20
GDM (%)	3.8	5.0	0.71
**Delivery outcomes**			
Gestational age (weeks)	39.0 (26.0, 41.7)	39.1 (32.9, 41.3)	0.93
Preterm (%)	9.9	11.5	0.80
Cesarean birth (%)	18.3	18.0	1.00
Birth weight (kg)	3.4 (0.6)	3.4 (0.6)	0.87
Macrosomic (%)	13.7	14.8	0.98

Means (SD), medians (range) and proportions are reported and Student’s t-test, Wilcoxon Rank Sum tests and Fisher’s Exact tests were performed, respectively. *NH* non-Hispanic, *BMI* body mass index, *GDM* gestational diabetes mellitus. *p < 0.05, **p < 0.01, p < 0.001.

Significant values are in [bold].

### Pregnancy and delivery outcomes

#### Intention to treat analysis outcomes

In the intention to treat analysis, although there are no differences in pregnancy and delivery outcomes between all pregnancy exercisers relative to non-exercisers (Table 1) or between all OWOB with NW women (Suppl. Table 1). On average, OWOB pregnant women exerted less weekly energy expenditure (442.3 vs 599.3 MET∙min∙week^−1^) during the pregnancy intervention relative to normal weight women (Suppl. Table 1); although not significant, 8% more overweight or obese pregnant women delivered via cesarean birth and 4.6% more delivered preterm compared to their normal weight counterparts (Suppl. Table 1). Intention to treat analysis of exercise group stratified by BMI category demonstrate significant differences in exercise level, aerobic capacity, such that OWOB exercisers had lower aerobic capacity, exercised less, and had higher rates of cesarean birth relative to the NW exercisers (Table 2).

**Table 2:** Intention to treat analysis of maternal descriptive, pregnancy and delivery outcomes by *Intervention and BMI Group.*

Characteristics	NW			OWOB		
	Exercise (n = 72)	Control (n = 26)	p value	Exercise (n = 59)	Control (n = 35)	p value
**Demographics**						
Age (year)	30.8 (4.1)	30.1 (3.5)	0.85	30.2 (3.7)	28.8 (4.5) ^**†**^	0.38
Parity	2.0 (1.0, 3.0)	1.5 (1.0, 2.0)	0.30	2.5 (1.0, 4.0)	2.0 (1.0, 3.0)	0.77
NH black (%)	9.2	11.5	0.66	16.1	36.4	0.01**
Pre-pregnancy BMI (kg m^−2^)	22.1 (1.7)	22.1 (1.4)	1.00	28.9 (3.6) ^**††**^	30.4 (4.4) ^‡^	0.09
Aerobic capacity (VO_2peak_)	25.2 (4.5)	23.6 (4.0)	0.37	21.3 (4.1) ^**††**^	20.0 (3.4) ^††, ¥^	0.56
Exercise level (MET∙min∙week^−1^)	693.8 (249.0)	359.4 (157.9)	** < 0.001**	589.7 (309.1) ^§§, ¥¥^	236.1 (169.0) ^**††**^	** < 0.001**
**Pregnancy outcomes**						
Gestational weight gain (lbs.)	31.9 (8.63	31.8 (11.0)	1.00	33.2 (15.0)	28.8 (13.5)	0.30
GDM (%)	2.8	8.0	0.59	5.1	2.9	0.33
**Delivery outcomes**						
Gestational age (weeks)	39.4 (34.0, 41.9)	35.5 (34.0, 41.3)	0.86	38.5 (24.0, 41.4) ^§^	39.2 (32.9, 41.3)	0.44
Preterm (%)	5.6	15.4	0.43	15.3	8.6	1.00
Cesarean birth (%)	11.1	23.1	0.21	27.1^§^	14.3	0.53
Birth weight (kg)	3.4 (0.4)	3.3 (0.5)	0.37	3.3 (0.7)	3.4 (0.7)	0.99
Macrosomic (%)	11.1	7.7	0.61	16.9	20	0.89

Means (SD), medians (range) and proportions are reported and Student’s t-test, Wilcoxon Rank Sum tests and Fisher’s Exact tests were performed, respectively. *NH* non-Hispanic. Within BMI class comparison *p < 0.05, **p < 0.01, p < 0.001; ^§§^p = 0.06 relative to NW Exercise group; ^§^p < 0.05 relative to NW Exercise group; ^**†**^p = 0.01 relative to NW Exercise group; ^**††**^p < 0.001 relative to NW Exercise group. ^‡^p < 0.001 within BMI and between NW BMI groups; ^¥^p < 0.01 relative to NW Control group; ^¥¥^p < 0.001 relative to NW Control group.

Significant values are in [bold].

#### Per protocol analysis outcomes

For per protocol analysis of those exercise adherent, OWOB exercisers were, on average, Caucasian, had a lower pre-pregnancy BMI (28.5 vs 30.4 kg m^−2^) and higher pre-pregnancy aerobic fitness (22.0 vs 20.0 ml O_2_∙kg^−1^∙min^−1^) relative to OWOB controls (Table 3). For per protocol analysis of those exercise adherent, significant between-group differences were observed for maternal age, race, pre-pregnancy BMI, and aerobic capacity where women in the exercise group were, on average, older (31.1 vs 29.4 years), Caucasian, had a lower pre-pregnancy BMI (24.6 vs 26.8 kg m^−2^) and higher fitness level (24.6 vs 21.6 ml O_2_∙kg^−1^∙min^−1^). Though not significant, exercisers have attenuations of 2.1% of cesarean births and 3% of preterm births relative to non-exercisers (Suppl. Table 2). For the per protocol analyses, a greater proportion of all OWOB pregnant women were non-Hispanic black (22.6 vs 8.5%), less aerobically fit (21.1 vs 25.3 ml O_2_∙kg^−1^∙min^−1^) and exerted less weekly energy expenditure (459.1 vs 626.1 MET∙min∙week^−1^). Although not significant, only 2.6% more OWOB women delivered via cesarean birth relative to the healthy BMI group; this is an attenuation of 5.4% from the intention-to-treat comparison (Suppl. Table 3).

**Table 3:** Per Protocol analysis of Maternal Descriptive, Pregnancy and Delivery Outcomes by *Intervention and BMI Group.*

Characteristics	NW			OWOB		
	Exercise (n = 51)	Control (n = 26)	p value	Exercise (n = 31)	Control (n = 35)	p value
**Demographics**						
Age (year)	31.4 (3.7)	30.1 (3.5)	0.14	30.6 (3.2)	28.8 (4.5) ^**†**^	0.06
Parity	2.0 (1.0, 3.0)	1.5 (1.0, 2.0)	0.30	1.5 (1.0, 2.0)	2.0 (1.0, 3.0)	0.22
NH black (%)	6.7	11.5	0.66	6.9	36.4	0.01**
Pre-pregnancy BMI (kg m^−2^)	22.3 (1.7)	22.1 (1.4)	0.65	28.5 (2.6) ^‡^	30.4 (4.4) ^‡^	0.03*
Aerobic capacity (VO_2peak_)	26.1 (4.2)	23.6 (4.0)	0.01**	22.0 (4.1)	20.0 (3.4) ^**††,** ¥^	0.045*
Exercise level (MET∙min∙week^−1^)	773.6 (29.2)	359.4 (39.3)	** < 0.001**	748.1 (38.6) ^¥¥^	236.1 (33.9) ^**††**^	** < 0.001**
**Pregnancy outcomes**						
Gestational weight gain (lbs.)	31.9 (8.6)	31.8 (11.0)	0.97	34.2 (14.8)	28.8 (13.5)	0.12
GDM (%)	3.9	8.0	0.59	9.7	2.9	0.33
**Delivery outcomes**						
Gestational age (weeks)	40.8 (34.0, 41.9)	35.5 (34.0, 41.3)	0.33	39.1 (34.0, 41.4)	39.2 (32.9, 41.3)	0.83
Preterm (%)	7.8	15.4	0.43	9.7	8.6	1.00
Cesarean birth (%)	11.8	23.1	0.21	22.6	14.3	0.53
Birth weight (kg)	3.4 (0.4)	3.3 (0.5)	0.37	3.4 (0.6)	3.4 (0.7)	0.99
Macrosomic (%)	5.9	7.7	0.65	6.5	20%	0.61

Means (SD), medians (range) and proportions are reported and Student’s t-test, Wilcoxon Rank Sum tests and Fisher’s Exact tests were performed, respectively. *NH* non-Hispanic, *BMI* body mass index, *GDM* gestational diabetes mellitus. Within BMI class comparison *p < 0.05, **p < 0.01, p < 0.001; ^**†**^p = 0.01 relative to NW Exercise group; ^**†**^ p < 0.001 relative to NW Exercise group. ^‡^p < 0.001 within BMI and between NW BMI groups; ^¥^p < 0.01 relative to NW Control group; ^¥¥^p < 0.001 relative to NW Control group.

Significant values are in [bold].

#### Regression analysis outcomes

Results for the effects of maternal BMI on pregnancy and delivery outcomes and the independent effect of prenatal exercise showed that, after controlling for maternal race, maternal BMI was not significantly associated with risk of non-elective cesarean birth (RR 1.63, 95% CI 0.92, 2.89) (Table 4). While the relationship remained non-significant, the inclusion of maternal exercise reduced the relative risk (RR 1.63–1.43) for the association of maternal BMI and risk of cesarean birth (Table 4). Further, after controlling for gestational age and maternal race, maternal BMI was positively associated with neonatal birth weight, where OWOB women delivered heavier neonates compared to normal weight pregnant women (β = 0.13, 95% CI 0.01, 0.25, p = 0.04). The independent effect of maternal exercise showed an increase in the difference in neonatal birth weights between OWOB pregnant women and their normal weight counterparts (β = 0.16, 95% CI 0.02, 0.29, p = 0.02), following its inclusion in the statistical model (Table 4b). Pre-pregnancy BMI (p = 0.004) and aerobic capacity (p = 0.03) are significant predictors of cesarean birth (Table 4b). Controlling for maternal age and aerobic capacity, GWG (p = 0.001), and race (p ≤ 0.0001) correlated with increased birth weight (Table 4b). Similarly, controlling for maternal age with exercise added to the model, lower aerobic capacity (p = 0.03), GWG (p = 0.002), Black race (p ≤ 0.0001) and exercise level (p = 0.03) correlated with birth weight (Table 4b). There were no significant prediction models for preterm birth.

**Table 4:** Linear and Poisson regression coefficients (β, 95% CI, p value) for the effects of prenatal exercise on overweight or obesity on cesarean birth and birth weight.

Characteristics	Model (BMI + covariate[s])			Adjusted (Model 1 + Exercise)		
	β or RR**	95% CI	p value	β or RR**	95% CI	p value
Cesarean birth**						
Overweight or Obese	1.63	0.92, 2.89	0.09	1.43	0.76, 2.72	0.27
**Birth weight (kg)**^**€**^						
Overweight or obese	0.13	0.01, 0.25	0.04	0.16	0.02, 0.29	0.02
Cesarean birth**			**0.01**			0.07
Prepregnancy BMI (kg m^−2^)	− 0.13	− 0.23, − 0.04	**0.004**	− 0.12	− 0.22, − 0.02	**0.02**
Aerobic capacity (VO_2peak_)	− 0.11	− 0.21, − 0.01	**0.03**	− 0.12	− 0.24, − 0.01	**0.03**
Exercise level (METmin week^−1^)				0.0005	− 0.0001, 0.002	0.51
**Birth weight (kg)**^**€**^			** < 0.0001**			** < 0.0001**
Maternal race (black)	− 0.54	− 0.77, − 0.31	** < 0.0001**	− 0.51	− 0.75, − 0.27	** < 0.0001**
Gestational weight gain (lbs)	0.01	0.004, 0.02	**0.001**	0.01	0.004, 0.02	**0.002**
Maternal age (years)	− 0.02	− 0.04, 0.0003	0.05	− 0.02	− 0.04, 0.001	0.07
Aerobic capacity (VO_2peak_)	− 0.02	− 0.03, − 0.002	0.08	− 0.02	− 0.04, − 0.002	**0.03**
Exercise level (METmin week^−1^)				0.0003	0.003, 0.0006	**0.03**

*Model-specific covariates included (1) cesarean birth: maternal race, MET∙min∙week^−1^; (2) birth weight: gestational age, maternal race, MET∙min∙week^−1^. **Poisson regression was performed, and the respective relative risks and 95% CIs were reported. ^€^Linear regression was performed and the respective βs and 95% Cis were reported. Normal weight served as referent group.

Significant values are in [bold].

## Discussion

The purpose of this study was to assess the influence of maternal exercise on the association of maternal BMI, cesarean birth, preterm birth, and neonatal birth weight. We hypothesized that prenatal exercise would decrease primary cesarean occurrence and preterm births and be associated with normal range birth weights in overweight and obese women, and thus, be similar to that of pregnancy outcomes in normal weight women. The major findings of this study were as follows: (1) maternal BMI was associated with risk of non-elective cesarean birth, (2) aerobic capacity predicted risk of cesarean birth, 3) while maternal exercise reduced the magnitudes of the relative risks, and 4) maternal exercise as well as aerobic capacity and GWG predict neonatal birth weight.

The present study found a significant relationship between maternal BMI and the risk of cesarean birth, which is consistent with current literature^22–24^. Previously, a significant positive correlation between maternal pre-pregnancy BMI and risk of cesarean birth at term has been shown^22,25^. It is possible that the increased risk for cesarean birth associated with OWOB may be due to additional comorbidities such as inflammation, and/or gestational diabetes, related to maternal BMI. Of note, BMI is calculated using only height and weight, thus, it is unable to determine whether increased weight is due to increased adipose tissue or increased lean mass. Given the former, it is suggested that future studies evaluate the association between specific body compartments, i.e., body fat, muscle mass, during pregnancy and the risk of cesarean birth delivery.

Interestingly, we found that early pregnancy aerobic capacity predicted the risk of cesarean birth, and although not significant, exercise during pregnancy reduced the magnitude of relative risk of cesarean birth rates. Previous research has demonstrated that fitness level, regardless of BMI, decreases inflammatory markers, i.e., C-reactive protein, associated with metabolic phenotype^26^. By reducing the amount of chronic inflammation at the beginning of pregnancy, during the development of the placenta, it is possible that OWOB women will be less likely to experience pregnancy and delivery complications associated with cesarean birth. Additionally, exercise has also been shown to improve placental development and function (i.e. blood flow and nutrient transfer)^27–30^. As seen in Table 3, the relative risk of cesarean birth decreased from 1.63 to 1.43 when prenatal exercise was included. Although this was not measured in this study, we speculate that this may be attributable to the effect exercise has on both placental function and overall maternal health. Along those lines, antenatal exercise has been shown to reduce inflammation^10,11,31^. Clapp et al. found that women who engaged in endurance exercise during pregnancy had lower levels of inflammation, as measured by tumor necrosis factor-alpha (TNF-α) concentration, compared to non-exercising women^10^. It was also reported that women who stopped exercising during their pregnancy had an increase in TNF-α, returning similarly to that of their non-exercising counterparts^10^. Another study reported lower increases in interleukin 1 beta (IL-1β), another marker of inflammation, with concurrent exercise training during pregnancy compared to controls^11^. In addition to potential decreases in inflammation during pregnancy and physiological adaptations to feto-placental development, it is possible that these changes are due to the overall effect on neonatal size. it is important to note that this itself may be the reason for the decreased risk of cesarean deliveries rather than physiological changes. Additional research is needed to examine how these physiological and/or anatomical improvements may be related to cesarean birth.

Lastly, we found that maternal exercise, aerobic capacity, and GWG predicted neonatal birth weight. It is plausible that fitness level, regardless of BMI, decreases inflammatory markers (i.e., C-reactive protein)^26^, thus improves placenta development and helps to normalize birth weight. Similar to previous studies, we also found that GWG was associated with birth weight^32,33^. Based on our previous findings demonstrating prenatal exercise participation is associated with decreased maternal insulin and lipid levels, we suspected that exercise in OWOB pregnant women would result in more birth weights within the normal range^6,34^. Interestingly, we found that exercise increased the birth weight differential when comparing OWOB and normal weight pregnant women. This could suggest that there is a differential impact of maternal exercise on normal weight versus overweight versus obese women. Further research should explore this area utilizing more specific measures (i.e., birthweight percentile, fat mass, muscle mass).

A major strength of this study is the randomized controlled design. Furthermore, it provided supervised exercise at the recommended level based on the American College of Obstetricians and Gynecologists. One potential limitation of the present study is that, due to small sample sizes, OWOB pregnant participants were combined into one group, preventing the analysis of individual BMI classifications (normal weight vs overweight vs obese). Although previous cesarean birth is a predictor for future cesarean birth, we did not have this data to control for in the multivariate analysis. Women with OWOB are at a higher risk for delivering small and large for gestational age infants, while women categorized as overweight are at risk for delivering large for gestational age babies^35^; thus, there may be significant physiological differences within the OWOB pregnant women that were masked due to inability to differentiate the groups. Further analyses considering maternal blood pressure and other outcomes (lipids and lipoproteins, etc.) with respect to this potential limitation is also suggested. Similarly, by collapsing these two BMI classifications into one group, it is possible that we diluted the effect of prenatal exercise, resulting in a greater birth weight difference between groups. Since we had a fairly healthy population (i.e. non-smokers, non-diabetics), this may limit the generalizability of our findings. However, further research is needed to determine if prenatal exercise impacts birth weight differently for pregnant women who are normal weight, overweight, or obese.

## Conclusion

In conclusion, prenatal exercise in women with OWOB during mid- to late-pregnancy resulted in a modest, but insignificant, reduction in the magnitude of relative risk of cesarean birth compared to normal weight counterparts. Additionally, prenatal exercise increased the differential in birth weights of neonates born from mothers with OWOB relative to the birth weights of neonates born to normal weight mothers. Importantly, aerobic fitness in early pregnancy influences delivery outcomes. These findings suggest that exercise before and during pregnancy may decrease the risk of cesarean birth in women with OWOB, potentially through changes in neonatal birth weight and improvement in other maternal health outcomes. Further research is needed to determine the physiological mechanism(s) of this relationship and whether women respond differently to prenatal exercise based on their BMI.

## Supplementary Information

Supplementary Information.

## Data Availability

Deidentified data may be made available upon request to Dr. Linda May, lead investigator.
