# Supplementary material for: Influence of prenatal exercise on the relationship between maternal overweight and obesity and select delivery outcomes
Source: Sci Rep. 2022 Oct 15;12:17343. doi: 10.1038/s41598-022-22283-0 (PMC9569361; doi:10.1038/s41598-022-22283-0)
Supplement: Supplementary file 1 — Supplementary Information. [file 41598_2022_22283_MOESM1_ESM.docx]

**Supplemental Table 1.** Intention-to-Treat analysis of Maternal characteristics by *Maternal BMI Classification*

| **Characteristics** | **OWOB**  **(n=94)** | **Normal Weight**  **(n=98)** | **p-value** |
| --- | --- | --- | --- |
| **Demographics** |  |  |  |
| Age (yr.) | 29.7 (4.0) | 30.6 (3.9) | 0.10 |
| Parity | 1.0 (1.0, 4.0) | 1.0 (1.0, 3.0) | 0.46 |
| NH Black (%) | 23.6 | 9.9 | **0.02*** |
| Aerobic Capacity (VO_2peak_) | 20.9 (4.0) | 24.8 (4.4) | **<0.0001***** |
| Exercise Level (MET∙min∙week^-1^) | 442.3 (312.6) | 599.3 (272.1) | **0.001***** |
| **Pregnancy Outcomes** |  |  |  |
| Gestational Weight Gain (lbs.) | 31.6 (14.5) | 31.9 (9.0) | 0.85 |
| GDM (%) | 4.3 | 4.1 | 1.00 |
| **Delivery Outcomes** |  |  |  |
| Gestational Age (weeks) | 38.8 (26.0, 41.4) | 39.3 (34.0, 41.7) | 0.19 |
| Preterm (%) | 12.8 | 8.2 | 0.35 |
| Cesarean birth (%) | 22.3 | 14.3 | 0.19 |
| Birth weight (kg) | 3.3 (0.7) | 3.4 (0.4) | 0.66 |
| Macrosomia (%) | 16.8 | 9.6 | 0.15 |

| Notes: Means (SD), medians (range) and proportions are reported and Student’s t-test, Wilcoxon Rank Sum tests and Fisher’s Exact tests were performed, respectively. NH = Non-Hispanic, GDM = gestational diabetes mellitus. *p<0.05, **p<0.01, ***p<0.001 |
| --- |

**Supplemental Table 2.** Per Protocol Analysis of Maternal Descriptive, Pregnancy and Delivery Outcomes by *Intervention Group*

| **Characteristics** | **Exercise**  **(n=82)** | **Control**  **(n=61)** | **p-value** |
| --- | --- | --- | --- |
| **Demographics** |  |  |  |
| Age (yr.) | 31.1 (3.5) | 29.4 (4.1) | **0.01*** |
| Parity | 1.0 (1.0, 4.0) | 1.0 (1.0, 3.0) | 0.73 |
| NH Black (%) | 6.8 | 25.4 | **0.003*** |
| Pre-Pregnancy BMI (kg/m^2^) | 24.6 (3.6) | 26.8 (5.4) | **0.01**** |
| Overweight or Obese (%) | 37.8 | 57.4 | **0.02*** |
| Aerobic Capacity (VO_2peak_) | 24.6 (4.6) | 21.6 (4.1) | **0.0002***** |
| **Pregnancy Outcomes** |  |  |  |
| Parity | 1.0 (1.0, 4.0) | 1.0 (1.0, 3.0) | 0.73 |
| Gestational Weight Gain  (lbs.) | 32.8 (11.3) | 30.0 (12.5) | 0.18 |
| GDM (%) | 6.1 | 5.0 | 1.00 |
| **Delivery Outcomes** |  |  |  |
| Gestational Age (weeks) | 39.0 (34.0, 41.4) | 39.1 (32.9, 41.3) | 0.64 |
| Preterm (%) | 8.5 | 11.5 | 0.58 |
| Cesarean birth (%) | 15.9 | 18.0 | 0.82 |
| Birth weight (kg) | 3.4 (0.5) | 3.4 (0.6) | 0.63 |
| Macrosomia (%) | 17.9 | 11.9 | 0.36 |

Note: Means (SD), medians (range) and proportions are reported and Student’s t-test, Wilcoxon Rank Sum tests and Fisher’s Exact tests were performed, respectively. NH = Non-Hispanic; BMI = body mass index; GDM = gestational diabetes mellitus. *p<0.05, **p<0.01, p<0.001

**Supplemental Table 3.** Per protocol analysis of Maternal descriptive, pregnancy, and delivery characteristics by *Maternal BMI Classification*

| **Characteristics** | **OWOB**  **(n=77)** | **Normal Weight**  **(n=66)** | **p-value** |
| --- | --- | --- | --- |
| **Demographics** |  |  |  |
| Age (yr.) | 29.7 (4.0) | 30.9 (3.6) | 0.06 |
| Parity | 1.0 (1.0, 4.0) | 1.0 (1.0, 4.0) | 0.09 |
| NH Black (%) | 22.6 | 8.5 | **0.03*** |
| Aerobic Capacity (VO_2peak_) | 21.1 (3.9) | 25.3 (4.3) | **<0.0001***** |
| Exercise Level (MET∙min∙week^-1^) | 459.1 (326.5) | 626.1 (279.5) | **0.002***** |
| **Pregnancy** |  |  |  |
| Parity | 1.0 (1.0, 4.0) | 1.0 (1.0, 4.0) | 0.09 |
| Gestational Weight Gain (lbs.) | 31.3 (14.3) | 31.9 (9.4) | 0.78 |
| GDM (%) | 6.1 | 5.3 | 1.00 |
| **Delivery** |  |  |  |
| Gestational Age (weeks) | 39.2 (32.9, 41.9) | 39.4 (34.0, 41.3) | 0.55 |
| Preterm (%) | 9.1 | 10.4 | 1.00 |
| Cesarean birth (%) | 18.2 | 15.6 | 0.82 |
| Birth weight (kg) | 3.4 (0.6) | 3.4 (0.5) | 0.67 |
| Macrosomia (%) | 21.5 | 10.0 | 0.07 |

| Means (SD), medians (range) and proportions are reported and Student’s t-test, Wilcoxon Rank Sum tests and Fisher’s Exact tests were performed, respectively. NH = Non-Hispanic; GDM = gestational diabetes mellitus. *p<0.05, **p<0.01, ***p<0.001. |
| --- |
